# Viral hemorrhagic fevers: advancing the level of treatment

**DOI:** 10.1186/1741-7015-10-31

**Published:** 2012-03-29

**Authors:** Giuseppe Ippolito, Heinz Feldmann, Simone Lanini, Francesco Vairo, Antonino Di Caro, Maria Rosaria Capobianchi, Emanuele Nicastri

**Affiliations:** 1 National Institute for Infectious Diseases Lazzaro Spallanzani, Via Portuense 292 00149 Rome, Italy; 2 Laboratory of Virology, Division of Intramural Research, National Institute for Allergy and Infectious Diseases, National Institutes of Health, Rocky Mountain Laboratories, 903 South 4th Street, Hamilton, MT 59840, USA; 3 Italian Health Cooperation, Piazzale della Farnesina 1 00135 Rome, Italy

**Keywords:** clinical management, innovative therapeutics, viral hemorrhagic fever

## Abstract

The management of viral hemorrhagic fevers (VHFs) has mainly focused on strict infection control measures, while standard clinical interventions that are provided to patients with other life-threatening conditions are rarely offered to patients with VHFs. Despite its complexity, a proper clinical case management of VHFs is neither futile nor is it lacking in scientific rationale. Given that patient outcomes improve when treatment is started as soon as possible, development and implementation of protocols to promptly identify and treat patients in the earliest phases of diseases are urgently needed. Different pharmacological options have been proposed to manage patients and, as for other life-threatening conditions, advanced life support has been proved effective to address multiorgan failure. In addition, high throughput screening of small molecular libraries has emerged as a novel promising way to find new candidates drugs for VHFs therapy and a relevant number of new molecules are currently under investigation. Here we discuss the current knowledge about VHF clinical management to propose a way to step up the approach to VHFs beyond the mere application of infection control measures.

## Background

The viral hemorrhagic fevers (VHFs) are a diverse group of animal and human illnesses that are caused by four distinct families of RNA viruses; these are: Arenaviridae, Filoviridae, Bunyaviridae, and Flaviviridae. The severity and clinical presentation of VHFs may significantly change according to several different factors related to the type of the causative agent, and the host epidemiological and clinical features. In general, patients with VHFs show fever and coagulation abnormalities that may progress towards disseminated intravascular coagulation, multiorgan failure, shock and, eventually, death. VHFs are always life-threatening diseases that may occur as isolated case(s), such as imported case(s) from endemic areas, or may cause devastating lethal outbreaks.

The management VHF cases has been mainly focused on the strict application of infection control measures to prevent transmission. However, high-level isolation and containment procedures may hamper the implementation of standard clinical interventions that would be offered to critically ill patients infected with other life-threatening pathogens. In developed countries, patients with septic shock are admitted to intensive care units to receive high-level primary medical and nursing care by a multidisciplinary healthcare worker (HCW) team. These patients have access to innovative medical drugs and to state-of-the-art therapeutics (sometime off label or under compassionate use protocols), and undergo any needed invasive intervention to control the infection and/or manage any complications that may arise. In contrast, when VHF is suspected, patients in similar conditions are usually confined to high containment isolation units [1-3]. In particular, in developing countries where VHFs are most common, confinement in combination with resource shortages may prevent patients receiving any adequate basic nursing and medical care. In fact, the recent *Médecins Sans Frontières *guidelines on the management of VHF patients in low-resource settings, though recognizing the issue of patient care, dedicate only a few lines to the clinical management issue [4].

Despite the practical difficulties of caring for VHF patients, a proper clinical case management of VHFs is neither futile nor does it lack of scientific rationale, as aggressive supportive care and specific antiviral therapy (whenever it is indicated) has been proved to improve patient outcomes.

Here we discuss current knowledge about experimental prophylaxis and treatment of VHFs to propose a way to step up the clinical approach to VHFs beyond the mere application of infection control measures.

## Discussion

### The clinical perspective for VHF patients

Beside high-level standards of safety for HCWs, which should be similar to those provided to bio-safety laboratory level 4 (BSL-4) personnel, a comprehensive protocol for clinical management of VHFs may not exclude high standards of care for patients. In theory patient care should include two main components. These are: (1) specific antiviral treatment (which includes compassionate or investigational use of therapeutic agents based on data from phase 1/2/3 trials, non-randomized studies or single case reports); and (2) advanced life support to prevent multiorgan failure.

In fact, in more routine circumstances (for example, bacterial sepsis), patient outcome depends on both targeted therapy (that is, antimicrobial therapy and modulation of host response) and the support of vital organ functions.

Based on clinical observations and on immunovirological parameters, the course of severe VHFs can be divided into three phases that require different clinical approaches; that is, incubation, precoagulopathy and coagulopathy [5]. The first phase begins with exposure to the virus and persists throughout the presymptomatic period. At this level, active/passive immunization and molecules with direct antiviral activity are the most effective pharmacological option. The second phase coincides with virus replication and the eventual viremia. At this stage molecules with direct antiviral activity can be still effective. The third and last phase is characterized by the onset of coagulation abnormalities driven by the dysregulation of the cytokine network (cytokine storm) [5,6]. At this stage immune-modulating drugs (for example, inhibitors of coagulation factors and/or proinflammatory cytokines) are the only sensible option although their actual efficacy is yet to be assessed.

### Case definition

Animal models and clinical experience in humans indicate that patient outcomes improve when treatment is started as soon as possible. Therefore, a case definition that can identify VHF patients in the early phases of diseases is urgently needed.

The World Health Organization proposed in 1999 a case definition for VHF to provide clinicians with a useful tool for early VHF detection [7]. However, as shown by Pittalis *et al. *[8] this case definition is too insensitive both for case finding during outbreaks and for early detection of suspected imported cases. In fact, the retrospective application of this case definition to 795 cases of Ebola VHF and 169 cases of Marburg VHF resulted in a sensitivity of 58.5% and 64.5%, respectively. This indicates that a revision of current case definitions for suspected VHF is necessary.

Since a universal case definition, valid in continuous surveillance for imported cases and during ongoing outbreaks, is hardly achievable, the best option is to have a broad-based, relatively unspecific definition based on both clinical (fever and other symptoms) and epidemiological (contact with a case) criteria. This definition may be promptly modified to include the specific characteristics of the particular circumstances to be faced, such as to confirm an imported cases in a developed country or the rapid response to prevent the transmission during a large ongoing outbreak in a low-resource setting.

### Laboratory diagnostics

Currently there are no commercial kits for diagnosis of VHFs, but a number of different diagnostics are available at specialized laboratories.

Nowadays, early diagnosis is mainly based on direct detection of viral antigens or RNA. In particular the United States Army Medical Research Institute of Infectious Diseases [9] and New York Columbia University [10,11], have recently developed multiplex PCR assays for specific detection of multiple VHFs. Although field data are limited (available only for Lassa), these systems have been evaluated for the detection of a consistent number of different agents form viral cultures, such as Ebola, Marburg, Lassa, Machupo, Junin, Sabia, Guanarito, Andes and Sin Nombre.

A further step to improve the molecular diagnosis of VHFs would be attained by developing assays using non-invasive sampling, which may be easier to obtain than blood/serum during outbreaks. Molecular assays using saliva or urine have been developed for dengue [12], Crimean-Congo hemorrhagic fever (CCHF) [13], Ebola [14] and there is evidence that a similar approach may be undertaken for yellow fever [15], Marburg [16] and Hantan [17] virus infections. However, it should be noted that blood/serum is the preferable diagnostic specimen for most VHFs.

Given the physiological kinetics of humoral response and the fact that VHFs are characterized by impairment of the antigen-presenting cell functions, antibody titers tend to be low at least in the earlier phases of diseases [6]. Therefore serology is usually not the primary diagnostic option in acute phase but can be particularly useful to confirm the diseases etiology in convalescent patients.

Resource shortages have always been the most relevant obstacles to the introduction of advanced diagnostics in low-income settings. However the experience of the 2005 Marburg outbreak in Angola [16] represented a model of how to face shortages of personnel and facilities even during outbreaks. On this occasion a mobile laboratory unit was deployed to provide quantitative real-time PCR assays with results available within 4 h since receiving a sample. The facility operated over a period of 88 days and tested 620 specimens from 388 individuals. It was possible to perform advanced diagnostics on different types of samples (including blood, oral swabs and milk from child-bearing women). The high concordance of test results from the mobile laboratory and those from the reference laboratory emphasizes that advanced field laboratory capacity should be made an essential part of future outbreak responses, as it is a relevant support tool for patient management and epidemiological surveillance.

### State-of-the-art of VHF treatment

Table 1 summarizes the main clinical and epidemiological features of different VHFs agents with specific recommended interventions to undertake.

**Table 1:** Treatment options for viral hemorrhagic fevers

Virus	Family	Incubation	Virulence	Communicability	Isolation	Treatment option(s)
Ebola	*Filoviridae*	2 to 21 days	High	Yes	Yes	Post-exposure vaccination (rVSV); rNAPc2; siRNA
Marburg	*Filoviridae*	2 to 21 days	High	Yes	Yes	Post-exposure vaccination (rVSV)
Lassa	*Arenaviridae*	6 to 21 days	Moderate	Yes	Yes	Ribavirin
Lujo	*Arenaviridae*	6 to 21 days	High	Yes	Yes	Ribavirin
Junin	*Arenaviridae*	6 to 21 days	Moderate	Yes	Yes	Passive immune therapy
Machupo	*Arenaviridae*	6 to 21 days	Moderate	Yes	Yes	Passive immune therapy
Sabia	*Arenaviridae*	6 to 21 days	Moderate	Unknown	Yes	
Guanarito	*Arenaviridae*	6 to 21 days	Moderate	Unknown	Yes	
Chapare	*Arenaviridae*	6 to 21 days	Unknown	Unknown	Yes	
Nipah	*Paramyxoviridae*	4 to 18 days	High	Potential^a^	Yes	Passive immune therapy
Hendra	*Paramyxoviridae*	4 to 18 days	High	Unknown	Yes	Passive immune therapy
CCHF	*Bunyaviridae*	3 to 7 days	Moderate	Yes	Yes	Ribavirin
Rift Valley fever	*Bunyaviridae*	2 to 14 days	Low to moderate	No	No	Ribavirin
Hantan, Seoul, and so on	*Bunyaviridae*	Days to months	Low to moderate	No	No	Ribavirinn, hemodialysis
Sin Nombre, Andes, and so on	*Bunyaviridae*	Days to months	Moderate	No^b^	No^b^	ECMO
Yellow fever	Flaviviridae	3 to 6 days	Moderate	No	No	
Dengue	Flaviviridae	3 to 14 days	Low	No	No	
RSSE	Flaviviridae	7 to 14 days	Moderate	No	No	
Omsk/KFD/AHF	Flaviviridae	3 to 8 days	Moderate	Unknown	Yes	

^a^Human-to-human transmission has been reported from outbreaks in Bangladesh.

^b^Andes virus has been reported to be transmissible from human to human; isolation may be indicated.

AHFV = Alkhurma hemorrhagic fever; CCHF = Crimean-Congo hemorrhagic fever; ECMO = extracorporeal membrane oxygenation; KFD = Kyasanur Forest disease; RSSE = Russian spring-summer encephalitis; rVSV = recombinant vesicular stomatitis virus; siRNA = small interfering RNA molecule.

### Active vaccination for post-exposure treatment

Active vaccination with a recombinant vesicular stomatitis virus (rVSV) expressing either the Zaire or Sudan ebolavirus glycoprotein 30 minutes after lethal challenge has shown 50% and 100% efficacy, respectively, in post-exposure treatment of rhesus macaques [18,19]. Similar results were obtained for a rVSV expressing the Marburg virus glycoprotein (strain Musoke) with five out of six and two out of six monkeys protected when the vaccine was given even 24 h or 48 h after lethal challenge, respectively [20]. This approach could be very useful in the scenario of an accidental exposure of a laboratory worker or HCW [21].

### Passive immune protection

Specific antibodies against different VHF agents are typically present 5 to 9 days after the onset of illness, and patients who die do not usually develop a measurable antibody response [22,23]. This finding encourages the therapeutic use of antibodies derived from recovered patients or animals. In fact, the potential benefits of immunoglobulin therapy were shown for treatments based on hyperimmune serum prepared from the blood of patients who recovered from CCHF or obtained by immunizing horses [24].

The transfusion of immune convalescent plasma with adequate doses of specific neutralizing antibodies is the current recommended treatment for Junín virus VHF; this intervention has been proved to be effective in improving clinical course of diseases and reducing mortality to less than 1% [25].

Bulgarian investigators reported the prompt recovery of seven severely ill patients treated with two specific immunoglobulin preparations for intramuscular and intravenous use, obtained from the plasma of CCHF survivors boosted with one dose of CCHF vaccine [26]. Similarly, seven out of eight patients who were infected during an outbreak in 1995 promptly recovered after transfusions of convalescent-phase blood from other subjects who had recovered from ZaireEbola virus infection [27]. In contrast, some non-human primate models for passive immune therapy, in particular for filovirus infections, are less encouraging. In fact, monkeys challenged with Ebola virus and eventually transfused with convalescent phase blood achieved high antibody titers but all succumbed to Ebola infection as quickly as control animals [28].

Besides conflicting results between empirical interventions and animal models for some VHFs, several issues should be considered when applying plasma therapy. Firstly, plasma therapy is relatively inefficient when initiated after 8 days of illness [25]. Secondly, a late self-limiting neurological syndrome has been observed in the 10% of patients who received convalescent plasma for Junín virus VHF. Thirdly, non-neutralizing crossreactive antibodies may potently exacerbate the disease in several conditions, such as Dengue and Marburg infections [29,30]. Fourthly, there is an inherent risk of transfusion-borne diseases. Fifthly, maintaining adequate stocks of plasma may be unfeasible. Finally, the neutralizing titers may decrease over time and plasma stocks must be periodically assessed prior to use.

### Antisense oligonucleotides

Gene-silencing treatment using small interfering RNAs (siRNA) has given good results both in guinea pigs [31] and non-human primate Ebola infection models [32]. In one experiment nine monkeys were challenged with Ebola virus, and subsequently three animals were treated with four doses of intravenous siRNA (at 30 minutes, and days 1, 3, and 5 post exposure), four animals with seven doses of intravenous siRNA (30 minutes and days 1 to 6 post exposure), and two animals were left untreated. Two of three monkeys who had received four post-exposure doses and all of those who had received seven post-exposure doses survived, while both controls monkeys died. This data provide evidence that RNA interference may be an effective post-exposure treatment strategy for humans infected with Ebola virus and, perhaps, other VHF agents. Unfortunately, production and cost issues significantly limit the use of this approach at present.

### Antivirals

Ribavirin is an antiviral drug currently recommended for prophylaxis and treatment of arenaviruses and bunyaviruses [33], in particular ribavirin has been used to treat patients with Lassa fever for more than 25 years [34,35]. In addition, a randomized double-blind placebo-controlled trial including 242 patients with serologically confirmed Hantan virus in the People's Republic of China found a sevenfold decreased mortality among ribavirin-treated patients [36]; however, other studies did not confirm these results. Ribavirin was also found to be effective against CCHF virus *in vitro *[37], but discordant results have been reported from treatment of CCHF patients in Iran and Turkey [38,39].

Pyrazinecarboxamide compounds, such as T-705 (favipiravir), T-1105 and T-1106, are being investigated *in vitro *and *in vivo *as candidate antiviral drugs for different VHFs. These compounds have shown good activity in animal models for arenaviruses (Junin virus), bunyaviruses, Rift Valley Fever virus (RVF), West Nile virus, and yellow fever virus [40].

FGI-106 is a new molecule that has shown broad-spectrum antiviral activity. FGI-106 showed potent *in vitro *activity against several lethal pathogens such as, Ebola, RVF and Dengue viruses. In addition in a mouse model of Ebola virus infection, FGI-106 was found to protect animals from lethal challenge [41].

High-throughput screening (HTS) of small molecular libraries, has recently emerged as a novel promising way to find new candidates molecules for VHF therapy [42]. An automated HTS system, integrated with optical microscopy, has been implemented to assess antiviral activity and potential cytotoxicity for chemical compounds against Dengue virus. This system assessed the antiviral activity against Dengue virus type 2 and cytotoxicity on human HEK293 of 5,632 molecules. In this way, 73 molecules were found to have both strong antiviral activity and no cytotoxicity and were identify as potential candidates for further *in vivo *research [43]. HTS technology has also been used on BSL-4 pathogens, such as filoviruses and arenaviruses, by the implementation of replication-incompetent virus pseudotypes. The research for arenaviruses discovered potent entry inhibitors against Lassa virus, Junin virus, Sabia virus, Machupo virus and Guaranito virus [42,44]. As well entry inhibitors for filovirus were also find by HTS approach. In particular a benzodiazepine derivative was identified with a 50% inhibitory concentration of 10 μM and 12 μM, for Ebola and Marburg viruses, respectively [45]. HTS has also been used to investigate molecules that may affect viral-host interaction. In particular an aminopyrrolidine compound (PF-429242) that acts as a potent SKI-1/S1P inhibitor (a cellular protease which is essential for the processing of viral envelop protein precursors) has recently been discovered. This compound has high stability, low toxicity and pharmacokinetic properties that made it an interesting drug candidate in the context of arenavirus [46,47] and CCHF virus [48] infections.

### Immune-modulating drugs

Several indirect lines of evidence suggest that modulating coagulation factors and/or cytokines activity may represent a new promising field of investigation for VHF therapy.

Firstly, studies on rhesus macaques demonstrate that therapy with recombinant inhibitor of factor VIIa/tissue factor and activated protein C are associated with significant increased survival after experimental infection with Zaire Ebola virus [49,50]. In addition recombinant human activated protein C showed good efficacy against severe malaria infections with multisystem organ failure [51]. Souza *et al. *[52] have also reported that activation of platelet-activating factor receptor (PAFR) could account for the major systemic manifestations of dengue infection. Activation of PAFR on endothelial cells and leukocytes induces increased vascular permeability, hypotension, and production of cytokines. In a mouse dengue model, treatment with an orally active PAFR antagonist (modipafant) prevented both the hypotension and the increase of vascular permeability, resulting in decreased lethality even when started 5 days after virus inoculation. This suggests that activated protein C, recombinant inhibitor of factor VIIa/tissue factor and modipafant might be considered in future clinical trials for severe dengue and/or Ebola infections in patients with shock not responsive to standard support treatments.

Secondly, preliminary studies have proposed a key role for macrophage migration inhibitory factor (MIF) in dengue pathogenesis [53]. MIF is a product of activated macrophages, which sustains macrophage survival and carries out its activity by suppressing p53-dependent apoptosis. Compared to wild-type mice, dengue-infected MIF -/- mice show decreased macrophage viability, decreased proinflammatory function and increased apoptosis, which results in lower viral load, less severe clinical manifestations and a significant delay in lethality. As patients with dengue hemorrhagic fever show elevated plasma concentrations of MIF [54], it is possible that neutralizing MIF activity may have a role in the treatment of severe dengue in humans.

Finally it was shown that transgenic mice expressing very high levels of human mannose-binding lectin concentrations (a C-type lectin that recognizes hexose sugars and acts as a first-line host defense against a wide range of viral pathogens) are more resistant than wild-type mice to fatal Ebola infections [55], this suggests that modulation of mannose-binding lectin activity may be an interesting field for further research.

### Advanced life support

Proper intensive care should be provided to all patients with VHF as for patients with other life-threatening conditions unrelated to VHF (for example, bacterial sepsis) [6]. Several clinical experiences from developed countries emphasize that an aggressive approach to address multiorgan failure in patients with Hantavirus VHF can be life saving. In particular an aggressive approach including steroids, vasoactive, hemodialysis and mechanical ventilation provided excellent outcomes in three patients (34, 42 and 72 years old) with multiorgan failure due to Puumala virus infection in Finland [56] and Austria [57]. In addition, retrospective data analysis of 38 subjects with severe Hantavirus cardiopulmonary syndrome in the USA [58,59] showed that initiation of extracorporeal membrane oxygenation to address acute respiratory distress syndrome may dramatically reduce complications and improve the overall survival. This confirms that invasive interventions, such as extracorporeal membrane oxygenation to address refractory hypoxemia [60] and hemodialysis to support renal function, should be considered in patients with VHFs.

### Potential application in the future

There is currently a need to develop standardized clinical protocols for VHF clinical case management that integrate infection control measures with comprehensive care for patients. In fact, although VHFs are most common in developing countries there are relevant strategic reasons for investing in them worldwide, given the increasing risk of exposure to VHF pathogens and the possibility of importation of VHF cases into non-endemic areas. In our opinion these protocols should be developed in two steps.

Firstly, protocols should be developed and assessed in developed countries on a limited number of patients. For this, 'centers of excellence' should be established. These centers should be able to provide: (1) safe, secure, high-quality and appropriate care, with optimum infection containment and control procedures [2]; (2) a high degree of expertise in critical care medicine and in the management of tropical diseases; (3) high containment (BSL-4) laboratories and skilled personnel to perform differential diagnoses [61]; (4) validated models for the management of such highly complex patients, including the timely access to investigational and compassionate use therapies; (5) established connections with air/ground transportation system(s) to transfer patients from the original location of exposure or the site where they enter the public health system to the treatment facility; and (6) established connections with international organizations and disease-oriented networks of infectious disease specialists and laboratories.

Drawing a simple scenario, the time between viral exposure and symptoms onset might be used to transport 'highly suspect' cases to a specialized center, fully equipped with all necessary infection control measures and clinical care expertise. At this center, assays for biochemical markers and differential diagnosis could be performed under adequate biocontainment measures and interventions could be undertaken under strictly controlled conditions. For example, the use of the experimental VSV-based Marburg vaccine could be justified up to 40 hours after exposure based on efficacy data in non-human primates and an expected slower rate of VHF disease progressions in humans than in macaques [62] and on the recent experience with the VSV-based Ebola vaccine following an Ebola virus needle-stick exposure in Hamburg [21]. In case of severely ill patients the transport should be carefully considered and a clinicoepidemiological oriented risk assessment should always be performed prior to making a decision on transport. Such an approach can help to establish and evaluate jointly shared and standardized protocols, applicable across developed countries which will serve as a model for other geographic regions, for the management of such rare but highly impacting infections.

The second, crucial step will consist in the translation of these protocols into a format suitable to be implemented in low-income settings. The main objectives will be to facilitate access to high-level standards of care, including experimental or compassionate use of therapeutics, to those who are in the greatest need. This kind of international cooperation for improving management of VHF patients is the most likely way forward for a better understanding and care of these rare and challenging clinical syndromes [63].

Support from national health authorities, major international public health partners and non-governmental organization would greatly facilitate the sustainability of the 'excellence centers' and the eventual production of protocols to be implemented in developing countries.

### Summary

Despite the obstacles to provide care for VHF patients, and the limited, even disappointing clinical data on the efficacy of clinically available drugs, we believe that providing access to promising new therapeutics, under compassionate use protocols in carefully controlled settings is not only ethically justified but urgently needed, given the high lethality of these diseases and the impact on national and international public health. In fact, even though VHF outbreaks are rare and proper isolation and high mortality during outbreaks will limit spread to neighboring communities (the outbreak will 'burn itself out'), this cannot justify the current limited access to the best available standards of care for the affected patients and exposed HCWs.

## Competing interests

The authors declare that they have no competing interests.

## Authors' contributions

GI and HF conceived the manuscript, decided the most topical issues to be discussed and coordinated coauthors. SL, EN and FV drafted the paper with regard to clinical management issues. MRC and ADC drafted the paper with regard to diagnostic issues. All authors read and approved the final manuscript.

## Pre-publication history

The pre-publication history for this paper can be accessed here:

http://www.biomedcentral.com/1741-7015/10/31/prepub
